# Factors Associated With Oral and Dental Health Utilization Among Individuals With Special Health Care Needs in Iran: A Cross‐Sectional Study

**DOI:** 10.1002/hsr2.73022

**Published:** 2026-08-19

**Authors:** Zahra Zare, Foad Iranmanesh, Mohammad Amin Bahrami, Fatemeh Shirazi, Zahra Kavosi

**Affiliations:** ^1^ Student Research Committee, School of Health Management and Information Science Shiraz University of Medical Science Shiraz Iran; ^2^ Department of Endodontics School of Dentistry, Rafsanjan University of Medical Sciences Rafsanjan Iran; ^3^ Health Human Resources Research Center, School of Health Management and Information science Shiraz University of Medical Science Shiraz Iran; ^4^ Department of Medical‐Surgical and Adult Nursing, School of Nursing and Midwifery Shiraz University of Medical Sciences Shiraz Iran

**Keywords:** Iran, oral and dental health, oral health, special health care needs, utilization

## Abstract

**Background and Aim:**

Individuals with special health care needs (SHCN) often encounter significant challenges in accessing oral and dental health services (ODHS), a problem that is even more pronounced in developing countries. This study aimed to identify the factors associated ODHS utilization among individuals with SHCN in Iran.

**Methods:**

This cross‐sectional study included 380 participants with SHCN, aged **≥** 5 years, residing in various districts of Shiraz in 2024. Data were collected through convenience sampling in rehabilitation centers, special education schools, and disability foundations. A researcher‐developed questionnaire was used for data collection based on existing surveys on oral health service utilization. Data analysis involved descriptive statistics (means, standard deviations, frequencies, and percentages), along with univariate and multivariate logistic regression using SPSS software.

**Results:**

Oral health problems were highly prevalent among the participants: 90% reported a history of toothache, and 72.4% had undergone at least one tooth extraction due to dental caries or abscess. Furthermore, 57.1% reported brushing their teeth before bedtime. Nearly one‐quarter of the participants (24.7%) had the highest level of dental care needs, while 67.1% reported unmet dental care needs, with treatment cost identified as the most commonly reported barrier (52.4%). Overall, only 33.7% had utilized oral and dental health services during the previous year. Multivariable logistic regression analysis indicated that place of residence, father's employment status, and maternal income were significant predictors of oral and dental health service utilization.

**Conclusion:**

ODHS utilization among individuals with SHCN was low, and unmet dental care needs were common. Financial barriers, particularly treatment costs, were frequently reported, and several socioeconomic factors were significantly associated with service utilization. These findings highlight the need for policies and planning aimed at reducing barriers to dental care access in this population.

**Abbreviations**
ASD: autism spectrum disorder
CI: confidence interval
CP: cerebral palsy
DD: developmental disability
DS: down syndrome
ID: intellectual disability
IDD: intellectual and developmental disabilities
ODHS: oral and dental health services
OR: odds ratio
SHCN: special health care needs

## 1 Introduction

Individuals with special health care needs (SHCN) encompass those with physical, developmental, mental, sensory, behavioral, cognitive, or emotional conditions that require medical management, healthcare interventions, and/or specialized support services [1]. According to statistics, around 1.3 billion people globally experience significant disabilities, accounting for approximately 16% of the global population [2]. Further, in the United States, the 2024 National Survey of Children's Health reported that one in five children—more than 14.5 million—have SHCN [3]. In Iran, based on the 2011 national census, approximately 1,017,659 individuals were reported to have disabilities, corresponding to an estimated prevalence of 13 per 10,000 population. Nevertheless, more recent nationwide disability statistics are not currently available in Iran [4].

These individuals demonstrate a higher demand for mental and physical healthcare services utilization than their counterparts without SHCN [5]. They encounter significant impediments within healthcare systems, adversely affecting their access to and experiences with medical care [6, 7]. These challenges include a wide range of issues from the complexities of securing a definitive diagnosis to the arduous task of navigating intricate care coordination processes as well as accessing requisite community support services [8].

In this context, oral health is a vital component of overall well‐being, playing a key role in supporting essential physiological functions owing to the fundamental role of the oral cavity [9, 10]. Those with SHCN encounter significant barriers in utilizing oral and dental health services (ODHS) [11]. Recent overview‐level evidence has indicated that these barriers are multifactorial and globally prevalent, covering financial constraints, provider‐related challenges, limited‐service accessibility, systemic healthcare issues, patient‐related factors, and concerns regarding the quality and continuity of care [12, 13].

Previous studies performed across different countries have further revealed that these barriers may vary according to healthcare system structures, professional preparedness, and local service availability. In many settings, physical accessibility constraints, financial burden, shortages of specialized services, and insufficient professional training have been identified as important factors influencing oral healthcare utilization among individuals with SHCN [14, 15]. Studies from Australia and the United States have additionally suggested that service delivery models and persistent barriers to preventive oral healthcare may affect both accessibility and quality of dental care [16, 17]. Likewise, evidence from Saudi Arabia suggests that limited clinical confidence, inadequate training, and insufficient resources among general dental practitioners may complicate provision of oral healthcare for this population [18].

Given the significance of oral health and the limited utilization of ODHS among individuals with SHCN, further research is needed to better understand the factors lined to ODHS utilization in this population. To the best of our knowledge, no cross‐sectional study has specifically examined these factors among individuals with SHCN in Iran. Accordingly, the present study aimed to explore the factors associated with ODHS utilization among individuals with SHCN in Iran.

## 2 Methods

### 2.1 Study Design and Setting

This cross‐sectional study was undertaken in Shiraz, Iran, from August 2024 to January 2025 to investigate factors associated with the utilization of ODHS among those with SHCN. Shiraz, the capital of Fars Province and one of the major metropolitan cities in southern Iran, hosts numerous health care, rehabilitation, and dental care centers serving a diverse population. The study was reported in accordance with the Strengthening the Reporting of Observational Studies in Epidemiology (STROBE) guidelines [19].

### 2.2 Participants

Individuals aged ≥ 5 years with at least one condition fulfilling the definition of SHCN were eligible to participate in this study. The participants who had sufficient cognitive ability completed the questionnaire independently. For those with limited cognitive ability, the questionnaire was completed with the assistance of a parent/caregiver or answered by the parent/caregiver on their behalf. Individuals who were uncooperative, healthy, or had conditions that did not significantly restrict access to ODHS, such as mild physical disabilities and elderly individuals, were excluded from the final analysis.

### 2.3 Data Collection

A stratified sampling approach was applied across rehabilitation centers, special education schools, and the Fars Province Disability Foundation. The sampling strata were defined based on the type of institution providing services to those with SHCN in order to include participants from different service settings. The eligible participants were identified with the assistance of center administrators and were consecutively invited to take part during the data collection period. A total of 520 eligible individuals were approached, of whom 410 agreed to participate (participation rate: 78.8%). Among those who consented, 384 completed the questionnaire. After excluding four questionnaires with substantial missing data, the final analytical sample consisted of 380 participants. Standardized procedures were employed along data collection to minimize reporting bias.

### 2.4 Study Instrument

The data collection instrument was a researcher‑developed questionnaire consisting of seven sections. The first section included 17 demographic questions related to individuals with SHCN as well as their families. The second section contained three questions appraising oral and dental hygiene behaviors, while the third section ascertained oral health status using two items.

The fourth section assessed oral health needs using 12 items measured on a five‑point Likert scale ranging from never (0), 1–2 times [1], sometimes [2], often [3], to every day [4]. Item scores were reverse‑coded prior to summation with lower total scores representing greater unmet oral health needs, while higher scores reflecting lower levels of need. The reverse‑coded item scores were summed to generate a total oral health need score ranging from 0 to 48.

Since no established clinical cut‑off values were available for this instrument, score categories were determined empirically using the quartile distribution of the total score within the study population. Based on this distribution, four categories were defined: high need (0–8), moderate‑to‑high need [9, 10, 11, 12, 13], average need [14, 15, 16, 17, 18, 19, 20], and low need [21] [48]. This scoring approach was employed to facilitate interpretation and comparison of oral health needs across participants.

The fifth section included three questions on the utilization of ODHS (at least one dental visit within the past 12 months (Yes/No) regardless of the reason for the visit), The sixth section consisted of eight items appraising access to and experiences with ODHS, and the seventh section included three questions related to unmet dental needs. The questionnaire was developed and administered in Persian. It was self‑administered whenever possible; nevertheless, when those with SHCN had limited cognitive ability, the questionnaire was completed with the assistance of a parent or caregiver or answered by them on the participant's behalf.

Content validity of the questionnaire was ascertained by 10 experts in healthcare management and dentistry. The questionnaire provided to the experts included a detailed explanation of the constructs being measured to ensure informed evaluation. The Content Validity Ratio (CVR) was calculated to determine the essentiality of each item. Items with a CVR value below 0.66 were eliminated. Further, the Content Validity Index (CVI) was calculated to evaluate item relevance and clarity. Items with CVI values between 0.70 and 0.79 were revised based on expert feedback, whereas items with higher values were retained.

Face validity was appraised by asking the experts to evaluate whether the questionnaire items were clear, understandable, and adequately reflected the main research questions. Experts also reviewed the wording, order, and comprehensibility of the items while also providing suggestions for improvement where necessary.

The reliability of the questionnaire was ascertained using the test–retest method. Thirty individuals with SHCN completed the questionnaire twice, with a two‑week interval between administrations. The test–retest reliability was calculated using the Pearson correlation coefficient, which yielded a value of 0.88, reflecting acceptable reliability. The participants involved in the reliability assessment were not included in the final study sample.

### 2.5 Study Variables

The outcome variable was ODHS utilization, defined as having at least one dental visit during the previous 12 months (yes/no), irrespective of the purpose of the visit.

Selection of independent variables was guided by previous literature and conceptually informed by the Andersen Behavioral Model of healthcare utilization [20]. Accordingly, variables were categorized into predisposing, enabling, and need‐related factors.

Predisposing characteristics included participant's age, sex, disability type, parental age, family size, birth order, family status, and parental education level.

Enabling factors consisted of household economic status, parental employment status, parental income, type of insurance coverage, and Supporting insurance.

Need factors were appraised using the oral health need score derived from questionnaire items assessing perceived oral health problems and treatment needs.

### 2.6 Statistical Analysis

Data were entered into Microsoft Excel and analyzed using IBM SPSS (version 27). Quantitative variables were reported as means and standard deviations, whereas qualitative variables were presented as frequencies and percentages.

Univariate and multivariate logistic regression analyses were undertaken to examine factors associated with the utilization of ODHS. The primary outcome variable was utilization of ODHS. Odds ratios (ORs) and 95% confidence intervals (CIs) were reported. A *p*‑value of less than 0.05 was considered statistically significant. All statistical tests were two‐sided.

The variables included in the multivariable logistic regression model were selected based on prior literature and their conceptual relevance within the Andersen Behavioral Model of health care utilization [20]. In the multivariate logistic regression analysis, variables were entered concurrently using the enter method. Economic status was excluded from the multivariate model owing to multicollinearity with parental income variables. Questionnaires with substantial missing data were excluded from the analysis, with analyses performed using complete‐case data.

The Hosmer–Lemeshow goodness‑of‑fit test was employed to assess the model fit. A non‑significant result (*p* > 0.05) indicated adequate model fit.

### 2.7 Ethical Consideration

This study, undertaken as part of a broader PhD research project, received ethical approval from the Ethics Committee of Shiraz University of Medical Sciences (Approval ID: IR.SUMS.NUMIMG.REC.1401.082). All methods were performed in accordance with relevant guidelines and regulations, including the ethical principles outlined in the Declaration of Helsinki. Written informed consent was obtained from parents, legal guardians, or accompanying caregivers when participants had limited cognitive capacity, and they were assured that all information would remain confidential. When those with SHCN had sufficient cognitive ability to understand the study procedures, informed assent—and when applicable, written informed consent—was obtained directly from them.

## 3 Results

### 3.1 Socio‐Demographic Characteristics

A total of 380 participants were included in this study. Table 1 reports the demographic characteristics of individuals with SHCN and their parents. The results indicated that the mean age of those with SHCN was 20.11 ± 9.23 years, and the majority (52.6%) were female. The mean age of fathers was 50.70 ± 10.13 years, while that of mothers was 46.43 ± 8.873 years. Also, the statistics revealed a mean family size of 4.23 ± 1.15 and a mean birth order of 1.98 ± 0.99 for them. Most families reported below‐average economic status on a 1–10 scale, with a mean score of 4.35 ± 2.14. Those with intellectual and developmental disabilities (IDD)—including Down syndrome (DS), autism spectrum disorder (ASD), cerebral palsy (CP), and intellectual disability (ID)—represented the largest proportion of participants (74.5%). Most individuals with SHCN lived with both parents (76.3%), and only 58.4% had supplemental insurance coverage.

**Table 1:** Socio‐demographic characteristics of individuals with SHCN.

Variables	Categories	No (%)
Gender	Boy	180 (47.4)
	Girl	200 (52.6)
Family status	Participant lives with both parents	290 (76.3)
	Single parent	68 (17.9)
	Parents are deceased	22 (5.8)
Disability type	IDD	283 (74.5)
	Sensory disability	35 (9.2)
	Multiple disabilities	62 (16.3)
Father's education level	Undergraduate	99 (26.1)
	Diploma	71 (18.7)
	Postgraduate Diploma & Bachelor	188 (49.5)
	Master & PhD	22 (5.8)
Mother's education level	Undergraduate	53 (13.9)
	Diploma	84 (22.1)
	Postgraduate Diploma & Bachelor	197 (51.8)
	Master & PhD	46 (12.1)
Father's employment status	Salaried employees	262 (68.9)
	Employer	22 (5.8)
	Unemployed	96 (25.3)
Mother's employment status	Salaried employees	78 (20.5)
	Employer	11 (2.9)
	Housewife	291 (76.6)
Father's income ($)	Without income	48 (12.6)
	< 97	93 (24.5)
	97–195	212 (55.8)
	> 195	27 (7.1)
Mother's income ($)	Without income	272 (71.6)
	< 97 $	47 (12.4)
	97–195 $	56 (14.7)
	> 195 $	5 (1.3)
Type of Insurance	Health insurance	118 (31.1)
	Tamin Ejtemaei	224 (58.9)
	Military insurance	18 (4.7)
	Komite Emdad	20 (5.3)
Supporting insurance	Yes	222 (58.4)
	No	158 (41.6)

### 3.2 Oral Health Status

Considering the oral and dental health status of the participants, the vast majority (*n* = 342, 90%) reported a history of toothache. Further, nearly three‐quarters of the study population (*n* = 275, 72.4%) had undergone at least one tooth extraction necessitated by dental decay or an abscess.

### 3.3 Oral and Dental Hygiene Behaviors

Table 2 presents oral and dental hygiene behaviors among individuals with SHCN. Approximately 57% of participants reported brushing their teeth before bedtime. Also, 30.5% brushed their teeth only once per day, and 15.8% reported brushing for less than 30 s.

**Table 2:** Oral and dental hygiene behaviors among individuals with SHCN.

Variables	Categories	No (%)
Do you brush your teeth before bedtime?	Yes	217 (57.1)
	No	163 (42.9)
How often do you brush your teeth during the day?	Less than once a day	20 (5.3)
	Once a day	116 (30.5)
	Twice a day	65 (17.1)
	Three times or more a day	16 (4.2)
How long do you usually brush your teeth?	< 30 S	60 (15.8)
	30 s–1 min	61 (16.1)
	1–2 min	58 (15.3)
	More than 2 min	38 (10)

### 3.4 Oral Health Need

According to the analysis, 29.5% of participants reported that along the past 3 months they sometimes experienced pain in their teeth, mouth, lips, or jaw, whereas 39.5% reported never experiencing such pain. Concerning gum bleeding, the majority of participants (79.2%) reported never experiencing it, while 11.8% indicated that it occurred sometimes. Further, 16.3% reported that food sometimes became stuck on the roof of the mouth, and 6.1% experienced this often.

With respect to sleep, 38.9% of participants reported sometimes having difficulty sleeping, whereas 36.1% had never experienced this problem. Additionally, 33.9% reported that the time they spent eating was never longer than that of others, while 31.1% stated that this occurred sometimes. Regarding sensitivity to hot or cold foods and drinks, 39.5% reported no difficulty, whereas 29.5% experienced this issue sometimes.

Also, 10.5% of participants reported sometimes missing school because of oral and dental problems. In terms of daily activities, 30% reported experiencing some degree of difficulty. Additionally, 32.6% stated that they sometimes did not want to talk to others, whereas 10.8% reported that this occurred often.

Concerns about health status were also common. Specifically, 28.4% sometimes worried that they were not as healthy as others, and 14.5% reported experiencing this concern every day. Likewise, 29.5% sometimes worried about being different from others, and 13.2% experienced this feeling on a daily basis. Ultimately, 25% of participants sometimes felt embarrassed about their condition, whereas 50.8% reported never feeling embarrassed (Table 3).

**Table 3:** Need for ODHS among individuals with SHCN.

In the past 3 months, how often have you had.?	Never	1–2 times	Sometimes	Often	Every day
	No (%)	No (%)	No (%)	No (%)	No (%)
Pain in their teeth, lips, jaw, or mouth	150 (39.5)	49 (12.9)	112 (29.5)	48 (12.6)	21 (5.5)
Gum bleeding	301 (79.2)	17 (4.5)	45 (11.8)	13 (3.4)	4 (1.1)
Food stuck on the roof of the mouth	259 (68.2)	19 (5)	62 (16.3)	23 (6.1)	17 (4.5)
Trouble sleeping	137 (36.1)	24 (6.3)	148 (38.9)	51 (13.4)	20 (5.3)
Eating longer than others	129 (33.9)	26 (6.8)	118 (31.1)	61 (16.1)	46 (12.1)
Difficulty eating or drinking hot or cold foods and drinks	150 (39.5)	30 (7.9)	112 (29.5)	50 (13.2)	38 (10)
Missed school due to oral health issues	307 (80.8)	24 (6.3)	40 (10.5)	5 (1.3)	4 (1.1)
Difficulty performing daily activities	134 (35.3)	15 (3.9)	114 (30)	49 (12.9)	68 (17.9)
Does not want to talk to other people	140 (36.8)	52 (13.7)	124 (32.6)	41 (10.8)	23 (6.1)
Worried about not being as healthy as others	147 (38.7)	17 (4.5)	108 (28.4)	53 (13.9)	55 (14.5)
Worried about differentiation from others	137 (36.1)	32 (8.4)	112 (29.5)	49 (12.9)	50 (13.2)
Embarrassed	193 (50.8)	36 (9.5)	95 (25)	43 (11.3)	13 (3.4)

**Figure 1:**
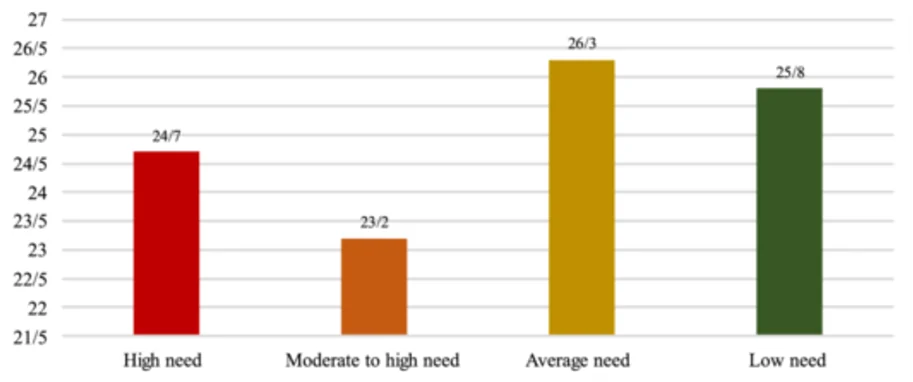
Scores of needs for ODHS among individuals with SHCN (percent).

Figure 1 demonstrates the distribution of need scores among individuals with SHCN. The total need score (range: 0–48) was calculated after reverse coding of the relevant items (Table 3), with lower scores representing higher levels of need. Based on the total score, the participants were categorized into four groups: high need (0–8), moderate‐to‐high need [9, 10, 11, 12, 13], average need [14, 15, 16, 17, 18, 19, 20], and low need [21] [48]. Overall, 24.7% of participants were classified as having high need, while 25.8% were categorized as having low need.

### 3.5 Dental Service Utilization

Table 4 outlines the utilization of ODHS among participants over the past year. Overall, 33.7% of individuals had visited a dentist, while 66.3% had not utilized any dental services along this period.

**Table 4:** Dental service utilization among individuals with SHCN.

Variables	Categories	No (%)
**Part A: Utilization (*n* = 380)**		
Visited a dentist in the past year	Yes	128 (33.7)
	No	252 (66.3)
**Part B: Visit frequency among users (*n*** = **128)**		
Number of dental visits in the past year	Once	44 (34)
	Twice	25 (20)
	Three times	16 (13)
	Four times	15 (12)
	Five times	4 (3)
	Six times	4 (3)
	Seven times or more	20 (16)
**Part C: Reasons for dental visit (multiple response; *n*** = **128)**		
Reasons for visiting a dentist in the past year	Dental Emergencies	9 (7)
	Restorative Services	58 (45)
	Surgical Procedures	52 (41)
	Orthodontic Treatments	9 (7)

Among those who sought dental care (*n* = 128), the majority reported infrequent use of services: 34% had visited a dentist once, 20% twice, and 13% three times in the past year. Only 6% reported seven or more visits.

With regards to the reasons for seeking dental care, restorative services were the most frequently reported (45%), followed by surgical procedures (41%). Dental emergencies and orthodontic treatments were less commonly cited, each accounting for 7% of reported reasons.

### 3.6 Access and Experience

Table 5 details the access characteristics and experiences related to oral and dental health services among individuals with SHCN. Among the study population, 50% reported usually utilizing private vehicles to access dental service centers, and 61.8% indicated that the waiting time for receiving services was typically less than 2 weeks. Further, 39.5% rated their experiences with dental services as normal.

**Table 5:** ODHS access and experiences among individuals with SHCN.

Variables	Categories	No (%)
Type of dental care facility used	Public centers	264 (69.5)
	Private centers	116 (30.5)
Travel time (min)	1–30	108 (28.4)
	30–60	185 (48.7)
	More than 60	87 (22.9)
Type of transportation	Walking	28 (7.4)
	Public transportation	162 (42.6)
	Private vehicle	190 (50)
Waiting time for emergency services (day)	< 1	86 (22.6)
	> 1	119 (31.3)
	No need for emergency services	175 (46.1)
Waiting time for routine services (week)	< 2	235 (61.8)
	> 2	145 (38.2)
Last dental appointment attendance	Yes	241 (63.4)
	No	94 (24.7)
	Other family	45 (11.8)
Evaluation of last dental visit	Perfect	45 (11.8)
	Very good	30 (7.9)
	Good	103 (27.1)
	Normal	150 (39.5)
	Bad	52 (13.7)
Dentist selection method	Friends or relatives	139 (36.6)
	Physicians	47 (12.4)
	Advertisements	7 (1.8)
	Randomly	98 (25.8)
	Others	89 (23.4)

### 3.7 Unmet Dental Needs

As presented in Table 6, 67.1% of individuals with SHCN reported that they needed ODHS but did not visit a dental center, indicating the presence of unmet dental needs. The participants reported several reasons for these unmet needs, with the most frequently cited being the cost of dental services (52.4%).

**Table 6:** Unmet dental needs.

Variables	Categories	No (%)
Unmet needs	Yes	255 (67.1)
	No	125 (32.9)
Causes of non‐utilization (Unmet needs)	Long distance to service delivery center	30 (7.9)
	Cost of services	199 (52.4)
	Low quality	33 (8.7)
	Fear and anxiety	57 (15)
	Clinic's operating days and hours	15 (3.9)
	Dentist– participant relationship	25 (6.6)
	Difficulty or impossibility of taking the participant to the dentist	57 (15)
	Self‐treatment	21 (5.5)
	Not a serious problem	32 (8.4)
	Others	13 (3.4)

**Figure 2:**
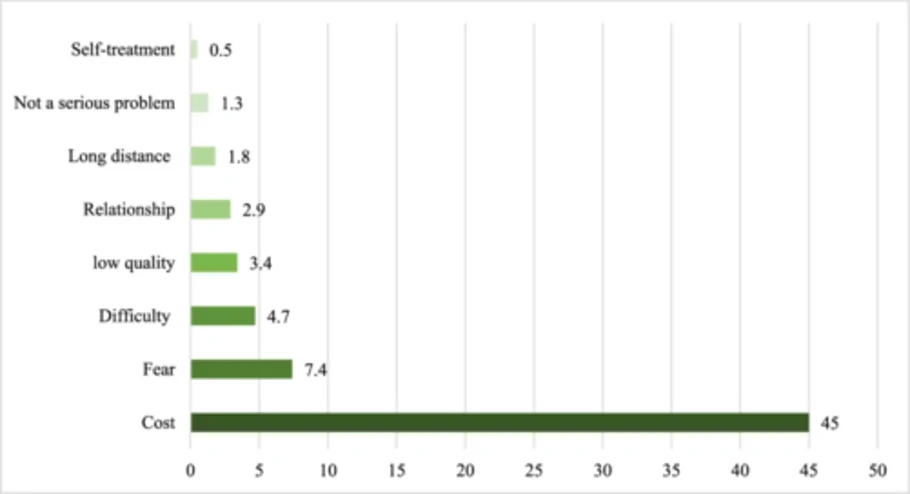
The main reason for unmet needs as perceived by individuals with SHCN (percent).

Figure 2 illustrates the prioritized primary reasons for not visiting dental service centers, highlighting the main factors contributing to unmet dental needs. While participants were allowed to select multiple reasons in Table 6, this figure depicts the single most important reason identified by each participant. The cost of dental services was the leading reason (45%), followed by fear (7.4%) and difficulty bringing individuals with SHCN to dental centers (4.7%).

### 3.8 Factors Associated With Utilization of ODHS

#### 3.8.1 Univariate Regression Analysis

The univariate logistic regression analysis revealed that family economic status was significantly associated with ODHS utilization (*p* = 0.03). Each one‑unit increase in economic status was linked to an 11.3% increase in the odds of ODHS utilization (OR = 1.11). Place of residence was also significantly associated with ODHS utilization (*p* = 0.007), as those living in urban areas had 4.34 times higher odds of using ODHS than those living in rural areas.

Father's employment status was significantly related to ODHS utilization (*p* = 0.009). Compared with individuals whose fathers were unemployed, those whose fathers had salaried jobs and those whose fathers were employers had 2.06‑ and 3.57‑times higher odds of utilizing ODHS, respectively. Further, both paternal and maternal income were significantly linked to ODHS utilization (*p* = 0.03 and *p* = 0.01, respectively). Individuals whose fathers earned between 97 and 195 USD per month or more than 195 USD per month had 2.16‑ and 4.09‑times higher odds of utilizing ODHS, respectively, compared with those whose fathers had no income. Conversely, maternal income less than 97 USD was correlated with a 60.5% reduction in the odds of ODHS utilization compared with no maternal income (OR = 0.39).

### 3.9 Multivariate Regression

Table 7 outlines the multivariate logistic regression results. Place of residence remained a significant predictor of ODHS utilization (*p* = 0.006), with urban residents exhibiting 5.06 times higher odds of using ODHS than rural residents.

**Table 7:** Multivariate analysis of factors accociated with utilization of ODHS in individuals with SHCN.

Variables	Adjusted OR	95% CI for OR adjusted		*p*‐value
		Lower	Upper	
**Father's age**	1.064	0.990	0.090	1.144
**Mother's age**	0.930	0.854	0.096	1.013
**Participant age**	1.013	0.970	0.552	1.058
**Number of family members**	1.155	0.872	0.315	1.530
**Birth order of the participant**	1.126	0.833	0.441	1.523
**Gender**				
Girl (ref)				
Boy	1.209	0.737	0.453	1.982
**Place of residence**				
Rural (ref)				
Urban	5.060	1.586	0.006	16.139
**Family status**				
Parents are deceased (ref)				0.328
Participant lives with both parents	2.017	0.645	6.310	0.228
Single parent	2.549	0.744	8.735	0.137
**Disability type**				
Multiple disabilities (ref)				0.649
IDD	1.353	0.662	2.765	0.407
Sensory disability	1.055	0.363	3.086	0.921
**Father's education level**				
Undergraduate (ref)				0.700
Diploma	1.069	0.477	2.395	0.871
Postgraduate Diploma & Bachelor	1.183	0.540	2.591	0.674
Master & PhD	0.541	0.119	2.459	0.426
**Mother's education level**				
Undergraduate (ref)				0.300
Diploma	2.283	0.885	5.890	0.088
Postgraduate Diploma & Bachelor	1.653	0.613	4.457	0.321
Master & PhD	1.217	0.308	5.805	0.779
**Father's employment status**				
Unemployed (ref)				0.017
Salaried employees	2.389	1.196	4.773	0.014
Employer	4.603	1.366	15.514	0.014
**Mother's employment status**				
Housewife (ref)				0.068
Salaried employees	3.263	0.712	14.948	0.128
Employer	10.947	1.451	82.588	0.020
**Father's monthly income (USD)**				
Without income (ref)				0.719
< 97	0.875	0.327	2.345	0.791
97–195	0.922	0.347	2.448	0.870
> 195	1.641	0.429	6.277	0.469
**Mother's monthly income (USD)**				
Without income (ref)				0.005
< 97	0.111	0.024	0.527	0.006
97–195	0.379	0.077	1.864	0.233
> 195	3.201	0.141	72.770	0.465
**Type of Insurance**				
Komite Emdad (ref)				0.897
Health insurance	1.272	0.405	3.991	0.680
Tamin Ejtemaei	1.221	0.406	3.670	0.722
Military insurance	0.811	0.157	4.192	0.803
**Supporting insurance**				
No (ref)				
Yes	1.359	0.820	0.234	2.254
**Need scores**				0.290
High need: 0–8 (ref)	1.102	0.542	2.237	0.789
Moderate to high need: 9–13	1.558	0.801	3.034	0.192
Average need: 14–20	0.814	0.412	1.608	0.553
Low need: 21–48				

Father's employment status also remained significant in the adjusted model. Compared with individuals whose fathers were unemployed, those whose fathers had salaried jobs and employer fathers had 2.38‑ and 4.60‑times higher odds of utilizing ODHS, (*p* = 0.01). Further, individuals whose mothers were employers had 10.94 times higher odds of utilizing ODHS than those whose mothers were homemakers (*p* = 0.02).

Maternal income remained significantly associated with ODHS utilization overall (*p* = 0.00). Compared with mothers without income, mothers earning less than 97 USD per month were linked to significantly lower odds of ODHS utilization (OR = 0.108, *p* = 0.00), corresponding to an 88.9% reduction in the odds of utilization.

The Hosmer–Lemeshow goodness‑of‑fit test demonstrated adequate model fit (*p* = 0.35). The model classification accuracy was 72.4%.

## 4 Discussion

In spite of considerable advancements in healthcare access and delivery, disparities in access and utilization remain in Iran [21], shaped by various factors ranging from socioeconomic status [22] to the availability of specialized care [23]. Such determinants are commonly conceptualized within Andersen's Behavioral Model of health care utilization [20], which describes healthcare use as the result of interactions between predisposing characteristics, enabling resources, and perceived need. In this respect, the present study explored factors associated with the utilization of ODHS, an important component of overall health among individuals with SHCN.

The results of this study revealed a significant gap between the ideal and current oral health status of those with SHCN in Iran. The high prevalence of cavities, abscesses, and tooth pain among participants highlights significant challenges in their oral health. This is in accordance with previous studies that have reported similar oral health issues among SHCN populations worldwide [24, 25, 26, 27].

Despite these findings, an assessment of their oral hygiene behaviors indicated that more than half of the participants brushed their teeth, suggesting a certain level of awareness and effort to maintain oral hygiene within these families. Nevertheless, the fact that a considerable portion of individuals brushed only once a day may suggest an association with insufficient oral hygiene practices. This may be related to inadequate knowledge about oral care or physical limitations imposed by their health conditions.

Likewise, Asiri et al. reported that nearly half of students with disabilities brushed irregularly and only a small proportion brushed twice daily or used dental floss. This highlighted generally suboptimal oral‑hygiene behaviors among individuals with disabilities and supports our findings [28].

In this context, a study undertaken in Jordan found that those with ASD had limited awareness of various aspects of oral health. Few individuals in the ASD group brushed their teeth once or twice a day [29]. Further, the study by Bernath et al. noted that individuals with ASD struggle to maintain oral hygiene owing to challenges related to their condition [30]. Also, other studies focusing on children with SHCN found that children whose caregivers worked in healthcare [31] and had higher levels of awareness [32] experienced better oral health and quality of life.

The other findings of this study underscore significant oral health challenges participants face, including gum bleeding and food sticking to the roof of the mouth. Gum bleeding is often a sign of periodontal disease, which, if left untreated, is linked to more severe oral health issues [33]. The experience of food sticking to the roof of the mouth may also reflect problems with oral or dental motor function, which may be associated with greater need for ODHS. A study in the United States found that the prevalence of gum bleeding in children with DD was more than twice that of typically developing children [34], supporting the findings of the present study. Further, several participants had difficulty eating various foods. In this regard, İslamoğlu et al. found that more than half of those with ID required assistance during meals, and many experienced issues such as choking, coughing, and a tingling sensation. They also discovered that those with severe chewing difficulties had higher DMFT scores, as also found by the current study [35].

The findings related to unmet dental needs among those with SHCN in this study are somewhat concerning. A high percentage of participants reported unmet needs in spite of recognizing the necessity of these services, suggesting the presence of fundamental barriers to accessing care. The cost of treatment, identified as the main reported reason for unmet needs, suggests financial constraints as a significant barrier to accessing dental services across vulnerable populations [36, 37].

This finding is in line with broader evidence indicating that children with intellectual disabilities often experience substantial challenges in fulfilling their healthcare needs due to systemic, social, and infrastructural barriers [38].

Fear and anxiety about dental services, particularly in children, have been associated with avoidance of dental visits, which may contribute to further oral health problems [39]. This issue may be more pronounced among those with SHCN, who are already confronting underlying challenges, potentially contributing to increased unmet dental needs. Moreover, the difficulty of transporting those with SHCN to dental care centers may further complicate access to care, especially for families with limited resources or individuals with mobility challenges, such as those with CP. Furthermore, the reported low quality of services suggests a need for improved dental care standards for the SHCN population, as a lack of trust in the quality of care may discourage individuals and families from seeking necessary services. This would potentially contribute to persistent unmet needs and poorer oral health.

These findings are also in accordance with a recent qualitative study, which highlighted the multifactorial nature of barriers affecting ODHS utilization among individuals with SHCN. That research identified financial constraints, insurance coverage, availability of specialized services, provider training, and parental awareness as key factors influencing access to dental care, emphasizing that both systemic and individual‐level determinants shape service utilization [40].

This study also found that fewer than one‐third of participants utilized dental services, with many individuals with SHCN lacking care. Significant barriers were linked to a lower likelihood of seeking essential dental care. In line with this, Alshatrat et al. found that factors such as embarrassment, lack of skilled and specialized providers, insufficient knowledge of how to treat individuals with disabilities, and inadequate facilities were reported as barriers associated with diminished access to dental care for individuals with ASD [41]. Another study also identified transportation difficulties, high dental treatment costs, and individuals' lack of cooperation with dentists as significant barriers to access and contributors to unfulfilled needs [42].

Meanwhile, the fact that many individuals who accessed dental services in the past year did so only once suggests gaps in continuous and comprehensive oral healthcare for this group. Moreover, the distribution of reported reasons for dental visits in the present study indicates that service usage was largely treatment‑oriented, with restorative and surgical procedures accounting for the majority of visits. This pattern may suggest that dental care among those with SHCN is often sought in response to existing problems rather than for preventive purposes. A study noted that half of individuals with disabilities had not visited a dentist for more than a year, and over 84% sought dental care only for emergency treatment. The study also reported that fear of the dentist was the most common barrier to dental care among those with disabilities, followed by cost, lack of cooperation, transportation challenges, distance, and dentists' lack of specialized skills [43]. In contrast to the present study's findings, another survey of individuals with ID found that more than half of the participants visited a dentist at least once a year [44]. This discrepancy may be linked to greater family awareness, lower treatment costs, or increased government support for these individuals.

Regression analysis results revealed that improved economic status was a significant predictor, emphasizing the importance of financial resources in accessing dental care. This finding accords with existing literature on the general population, underscoring the role of socioeconomic status in healthcare utilization [45, 46]. However, its impact is even more pronounced in vulnerable groups, as their access to care is more limited, and their families tend to have lower economic status.

Additionally, parental employment status and mothers' income were identified as influential factors, reinforcing the role of financial contributions and economic stability in parents' decisions regarding the dental care of individuals with SHCN. This finding may reflect the significance of household economic empowerment and the assurance of sufficient resources to support the dental needs of these individuals [12].

Compared to rural areas, urban residence was another significant predictor, highlighting disparities in ODHS between urban and rural environments. Urban areas have generally better accessibility to healthcare facilities, including dental services, a finding also supported by Obeidat et al. [30]. Another study revealed that children in urban areas were 1.62 times more likely to use dental services than those in rural areas. However, this gap has narrowed over time with regional development [47]. It is essential to address these geographic disparities through improved healthcare infrastructure and services in rural areas for ensuring equitable access to oral healthcare.

### 4.1 Limitations

This study had certain limitations. First, its cross‐sectional design restricts establishing causality between the identified factors and ODHS utilization. Further, the sample was drawn from a specific population in Iran, which may limit the generalizability of the findings to other regions or countries. Also, the participants were recruited through rehabilitation centers, special education schools, and disability support institutions. Although these settings provide access to a broad population of individuals with SHCN, those who were not affiliated with such centers—particularly those facing more substantial barriers to healthcare access—may have remained underrepresented in the study. In addition, exploratory or confirmatory factor analysis was not performed to further ascertain the construct validity of the questionnaire.

In spite of these limitations, the study provides valuable insights into the extent of dental service utilization among a vulnerable population and the factors influencing it. These findings can inform the development of targeted interventions and evidence‐based policies to ameliorate oral and dental health in this group. They also serve as a foundation for future research with more robust study designs and diverse samples to better understand the determinants of ODHS utilization across different populations.

## 5 Conclusion

This study emphasizes substantial oral and dental health challenges among those with SHCN in Iran. Even though a proportion of participants reported engaging in routine oral hygiene practices, the reported frequency and duration of toothbrushing suggest that oral hygiene behaviors remain suboptimal for many individuals. Dental problems, including cavities, gum bleeding, and toothache—were also highly prevalent in this population. Major barriers to the utilization ODHS included financial constraints, fear and anxiety, transportation difficulties, and concerns regarding the quality of care. Economic status, urban residency, and specific parental characteristics were identified as significant factors linked to ODHS utilization. These findings highlight the need for comprehensive and context‑sensitive strategies to address the multiple barriers to oral and dental health among individuals with SHCN. Strengthening access to services, ameliorating caregiver education and support, and developing targeted preventive programs may contribute to mitigating disparities as well as improving oral and dental health outcomes in this vulnerable population.

## Author Contributions

**Zahra Zare:** conceptualization, investigation, methodology, validation, visualization, writing – review and editing, writing – original draft, software, formal analysis, data curation, supervision, resources. **Foad Iranmanesh:** supervision, writing – review and editing, writing – original draft. **Mohammad Amin Bahrami:** supervision, writing – review and editing, writing – original draft. **Fatemeh Shirazi:** writing – original draft, writing – review and editing. **Zahra Kavosi:** conceptualization, investigation, funding acquisition, writing – original draft, writing – review and editing, visualization, validation, formal analysis, project administration, resources, supervision.

## Ethics Statement

This study, undertaken as part of a broader PhD research project, received ethical approval from the Ethics Committee of Shiraz University of Medical Sciences (Approval ID: IR.SUMS.NUMIMG.REC.1401.082). All methods were performed in accordance with relevant guidelines and regulations, including the ethical principles outlined in the Declaration of Helsinki. Written informed consent was obtained from parents, legal guardians, or accompanying caregivers when participants had limited cognitive capacity, and they were assured that all information would remain confidential. When those with SHCN had sufficient cognitive ability to understand the study procedures, informed assent—and when applicable, written informed consent—was obtained directly from them.

## Conflicts of Interest

The authors declare no conflicts of interest.

## Transparency Statement

All authors have read and approved the final version of the manuscript. The corresponding author (Z.K.) had full access to all of the data in this study and takes complete responsibility for the integrity of the data and the accuracy of the data analysis. Also, ZK affirms that this manuscript is an honest, accurate, and transparent account of the study being reported. All relevant aspects of the study, including the methodology, data collection, analysis, and interpretation of the results, have been described in sufficient detail to ensure clarity and reproducibility. No important aspects of the study have been intentionally omitted. Any deviations from the study as originally planned have been clearly stated and justified within the manuscript. The authors confirm that the data presented are authentic and that the study has been conducted and reported in accordance with accepted standards of scientific integrity and ethical research practice.

## Data Availability

The data that supports the findings of this study are available in the supporting material of this article.
